# Neglected tropical diseases activities in Africa in the COVID-19 era: the need for a “hybrid” approach in COVID-endemic times

**DOI:** 10.1186/s40249-020-00791-3

**Published:** 2021-01-04

**Authors:** David Molyneux, Simon Bush, Ron Bannerman, Philip Downs, Joy Shu’aibu, Pelagie Boko-Collins, Ioasia Radvan, Leah Wohlgemuth, Chris Boyton

**Affiliations:** 1 Department of Tropical Diseases Biology, Centre for Neglected Tropical Diseases, Liverpool School of Tropical Medicine, Pembroke Place, Liverpool, L3 5QA UK; 2 Neglected Tropical Diseases, Sightsavers, Airport, P O Box KIA18190, Accra, Ghana; 3 Neglected Tropical Diseases (Ascend West and Central Africa), Sightsavers, 35 Perrymount Rd, Haywards Heath, RH16 3BZ UK; 4 Neglected Tropical Diseases, Sightsavers, 23 Beverly Dr., Durham, NC 27707 USA; 5 Programme Operations, Sightsavers, Nigeria Country Office-No 1 Golf Course Road, P.O. Box 503, Kaduna, Nigeria; 6 Neglected Tropical Diseases, Sightsavers, Benin Country Office-3rd Floor of the Riveria Golf Building, Akpakpa, Lot 4002 F, Old Bridge, Republic of Benin; 7 Neglected Tropical Diseases, Sightsavers, 35, Perrymount Rd, Haywards Heath, RH16 3BZ UK; 8 M&C Saatchi World Services, 36 Golden Square, London, W 9EE UK

**Keywords:** COVID-19, Neglected tropical diseases, Mass drug administration, Morbidity management, Communities, Water, Sanitation and hygiene, Behaviour change

## Abstract

With the coronavirus disease 2019 (COVID-19) pandemic showing no signs of abating, resuming neglected tropical disease (NTD) activities, particularly mass drug administration (MDA), is vital. Failure to resume activities will not only enhance the risk of NTD transmission, but will fail to leverage behaviour change messaging on the importance of hand and face washing and improved sanitation—a common strategy for several NTDs that also reduces the risk of COVID-19 spread. This so-called “hybrid approach” will demonstrate best practices for mitigating the spread of severe acute respiratory syndrome coronavirus 2 (SARS-CoV-2) by incorporating physical distancing, use of masks, and frequent hand-washing in the delivery of medicines to endemic communities and support action against the transmission of the virus through water, sanitation and hygiene interventions promoted by NTD programmes. Unless MDA and morbidity management activities resume, achievement of NTD targets as projected in the WHO/NTD Roadmap (2021–2030) will be deferred, the aspirational goal of NTD programmes to enhance universal health coverage jeopardised and the call to ‘leave no one behind’ a hollow one. We outline what implementing this hybrid approach, which aims to strengthen health systems, and facilitate integration and cross-sector collaboration, can achieve based on work undertaken in several African countries.

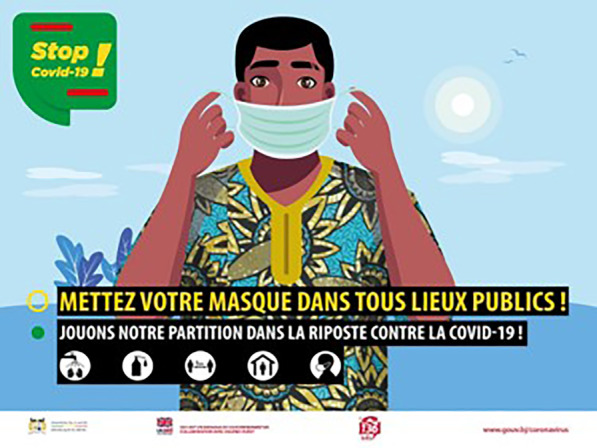

## Background

We have described the world as being “assaulted by COVID-19” [1]. The term ‘post-COVID-19’ has been used to provide assurance the assault will end, the impact will be temporary, and a post coronavirus disease 2019 (COVID-19) world will emerge. It is now clear, however, that COVID-19 is endemic globally and that the desired world will not emerge in the foreseeable future in the absence of a vaccine as effective as smallpox or polio although there is current optimism that a vaccine may be efficacious. Even with an effective vaccine we will need to learn to live with COVID-19. At the time of writing, Europe is experiencing a ‘second spike or surge’ of infection with similar resurgence patterns in many countries, albeit on different scales [2]. While the situation in some countries, like India and Latin America are of increasing concern, in the African continent COVID-19 appears to be at less of a public health risk than feared although as pointed out by the WHO African Regional Director health services are being impacted with declines in malaria treatment, skilled attendance at births and outpatient consultations as measured in 14 African countries [3]. There was a 50% reduction in the services World Health Organization Regional Office for Africa (WHO AFRO) monitored; over 1.3 million missed their first measles vaccination and in Nigeria 360 000 women missed ante-natal care and the number of maternal deaths in health facilities doubled compared with 2019 [3].

While we are hopeful that recent trends in Africa continue to stabilise and decline, global models have underlined the potential and disproportionate health impact of COVID-19 on low and middle income countries (LMIC) [4]. The longer-term economic implications for Africa have also been analysed in a United Nations Conference on Trade and Development (UNCTAD) report [5] (https://unctad.org/en/PublicationsLibrary/aldcmisc2020d3_en.pdf) which predicts a reduction of 1.4% of gross domestic product (GDP), with the countries with smaller economies being more severely impacted:

“Mainly a result of export adjustments affecting primary commodity exporters, and the attendant losses to tax revenue which reduce the capacity of government to extend public services necessary to respond to the crisis. Overall, this paper estimates a regional average of about 5% in public revenue losses in Africa, with total merchandise exports contracting by about 17%.”

Clearly, social consequences because of recessionary forces have the potential to adversely affect the most vulnerable. The grouping of diseases under one NTD umbrella was developed because of the low level of resource provided for conditions which disproportionately affect “the bottom billion”, given only 0.6% of Official Development Assistance (ODA) was devoted to interventions addressing these conditions, estimated to be afflicting up to two billion people [6, 7]. As it has been predicted that COVID-19 will enhance global inequity particularly in LMICs, it is essential that neglected tropical disease (NTD) programmes, with recognised success over the past decade, remain on the health and development agenda given their key role in promoting many of the Sustainable Development Goals [8]. Ehrenberg et al. [9] have recently summarised the relevant strategies that can support the control of the neglected tropical diseases in the context of the COVID-19 challenge with a focus on the zoonotic origins of the virus and the need for multisectoral approaches, exemplifying how synergies can be achieved to further efforts against NTDs.

The speed of viral spread across the globe suggests the fundamental biological characteristics of severe acute respiratory syndrome coronavirus 2 (SARS-CoV-2) are the same everywhere. It is biologically unlikely that in Africa SARS-CoV-2 has or will change its characteristics of infectivity, modes of transmission, or spectrum of clinical presentations—from asymptomatic at one extreme through to serious, life-threatening and fatal outcomes on the other whilst displaying the capacity for the virus to mutate and cross and re-cross the species barriers as shown in mink farms in Denmark [10].

The true prevalence of COVID-19 globally is likely to be much higher than published data, as reported by model analysis [11]. Given the varied quality of surveillance, the extent and effectiveness of testing, the accuracy of certification of the cause of death (if any) as well as the paucity death reporting, particularly in rural settings, the true situation is not known despite the suggestion, that to date, COVID-19 has not impacted on Africa as might have been expected or predicted [1, 3]. As the virus is now endemic COVID-19 mitigation strategies will therefore become the new normal for all medical interventions and consultations as well as public health programmes, including NTD programmes. Daily new global confirmed cases are around 300 000, with India and the USA representing most of the new cases, followed by Brazil, Mexico and other South American countries but significant spikes throughout Europe have generated “lockdowns” of varying degrees of severity and duration. The northern hemisphere winter period is also creating a spike in the pandemic in Europe and the USA. The Johns Hopkins Coronavirus Resource Center provides the real time country global updates [2]. While confidence in case reporting remains a concern, a plausible explanation for why Africa has apparently seen a greater success in slowing the spread of the virus is due to a greater adherence to the recommended preventative practices. There are also fewer super-spreader events documented. Such events are fuelled by increased travel during holiday periods and crowding in indoor settings like bars, restaurants, and indoor stadiums. Africa also has a proportionately larger rural community than other geographies—an advantage as most cases and local transmission have been primarily confined to urban areas. The median age of African populations is significantly lower than in Europe as pointed out in [1] at around 20 in Africa and 45 in Europe. Given most COVID-19 deaths are occurring in the post 65 cohort then the case fatality in Africa would be expected to be lower.

## Neglected tropical disease activities

Given the pandemic status of COVID-19, the response must be global. Countries should be learning from the experiences of others. Much is often made of what LMIC can learn from health services and systems in high-income countries but the response to the pandemic is an opportunity to re-address this flow of experience. Africa has well developed community health programmes that deliver at scale while engaging with their communities. As outlined above, NTD programmes have utilised the health messaging and behaviour change work that formed part of their portfolio to develop clear COVID-19 messaging. Crisp [12] highlights, for example, that we should deal with health in rich and poor countries in the same way, not treating them as totally different, and suggests that instead of talking about international development we should talk about co-development where learning goes both ways; he adds community health programmes in Africa developed a community-based and directed approach to the treatment of NTDs—the principles of which are relevant to any health setting [12].

Any protracted suspension of NTD activities, particularly MDA, will not only have a detrimental impact by enhancing the risk of NTD transmission, but will fail to leverage behaviour change messaging on the importance of hand and face washing and improved sanitation—a common strategy for several NTDs that also reduces the risk of COVID-19 spread [1, 13]. It also misses the opportunity for NTD programmes to mitigate the spread of the virus amongst health workers, while building resilience in responding to future emerging infectious diseases and to alleviate possible secondary health impacts thwarting progress to universal health coverage (UHC) [1, 13].

School-based NTD delivery programmes form an important part of NTD distribution strategies. While school openings have been staged over time in response to reduced COVID-19 risk, the large gathering of students and teachers increases the risk of transmission and enhances wider virus dissemination. If a policy decision has been made by national authorities to open schools it would seem logical for those involved with NTD MDA that distributing treatments in schools via social distancing methods can also reinforce COVID-19 prevention messages while house-to-house community distribution ensures children withheld from school are covered as well.

We consider that the full resumption of NTD interventions will not increase the risk of spreading SARS-CoV-2 given the use by government of risk assessment tools to recognise and mitigate risk using agreed standard operating procedures (SOPs), particularly when governments have made the decision to ease lockdown constraints, open schools, resume other health services, allow movement via transport, and permit mass gatherings to take place.

NTD programmes have long acknowledged the need to work across borders to control and eliminate diseases. The African Programme for Onchocerciasis Control (APOC) first started to advocate for this approach in 1995 which was been continued by the Expanded Special Project for the Elimination of NTDs (ESPEN) through, for example, cross-border disease maps on the ESPEN data base [16]. The examples of cross-border working on NTDs are numerous. One example is the case of Sierra Leone, Liberia and Guinea (Conakry) working on onchocerciasis under the banner of the Mano River Union from 2005 whereby coordination of treatment of border areas was conducted [17]. The management of cross-border, be they internal or international borders, is one of the most complex development issues to manage [18] but if taken seriously at the beginning of the planning stage and throughout implementation the mechanisms required can be utilised not only for NTDs but other health interventions. Sierra Leone, for example, in the COVID-19 pandemic has been able to pivot the structures on NTDs in the border areas in terms of community sensitisation. Ghana has set up community information points on the Ghana-Togo border and to train border community health staff on disease control measures.

Given the mitigation strategies being proposed by national NTD programmes as precautionary measures (e.g. facial covering for all community drug distributors, intensified handwashing practices, maintenance of physical distancing practices, etc.), the commencement of NTD programmes will have minimal risk in spreading the virus. The NTD platforms provide the opportunity to actively reinforce COVID-19 prevention practices via community distributors who have the influence and respect of their communities often distant from formal health facilities [14]. The continued suspension of MDA is not logical against this background. The NTD Modelling Consortium has projected the impact of suspension of MDA on transmission and the consequential delay in achieving the targets set in the new WHO/NTD Roadmap [15]. The results indicate that interruption of MDA activities will have varying impacts on established targets depending on the duration of any treatment delays, the specific disease targeted, initial baseline prevalence and the history of effective MDA, in particular, coverage. However, the ability of national governments to retain high levels of “enforcement”/lockdown has probably reached a limit in terms of social acceptability given the economic impact. Governments across the globe are being pressed to ease the critical and necessary public health restrictions associated with lockdown primarily for economic and social reasons, in fear of greater economic damage but also because of potential social unrest as is currently occurring in Lagos, Nigeria. However, in the northern hemisphere it is clear the second surge or spike commenced in late September/early October.

## Ascend programme and response to COVID-19: “flexing” towards a “hybrid” approach

The Foreign, Commonwealth and Development Office supports the *Ascend* programme led by Sightsavers in partnership with The Schistosomiasis Control Initiative Foundation, the Centre for Neglected Tropical Diseases, Liverpool School of Tropical Medicine, M & C Saatchi and Mott MacDonald and with the Ministries of Health of partner governments. In response to COVID-19 two approaches have been the basis of the response (1) risk mitigation methodologies and processes to maximise safety and reduce COVID-19 transmission risk to allow countries to decide on the resumption of NTD activities, and (2) ascend and partners have created appropriate country and sub-national specific health messaging through billboards, radio and TV slots in 11 countries. The examples of the materials developed, tested and displayed in the figures below. The rapid deployment of this educational and communication material to enhance adherence to the necessary behaviour change was facilitated by the existing Ascend NTD programme which adapted or “flexed” its resources working with national Ministries and local partners.

## Introduction to risk assessment mitigation action tools in the context of COVID-19

In July, the World Health Organization (WHO) provided interim guidance on Considerations for implementing mass treatment, active case‐finding and population-based surveys for neglected tropical diseases in the context of the COVID-19 pandemic [19]. This guidance proposed a two part decision-making framework: (1) a risk–benefit assessment, to decide if the planned activity should proceed; and (2) an examination of a list of precautionary measures that should be applied with the aim of decreasing the risk of transmission of COVID-19 associated with the activity, and strengthening the capacity of the health system to manage any residual risk.

In following WHO’s additional guidance on *adjusting public health and social measures in the context of COVID-19* [20] (16 April 2020), countries that adapt public health and social measures (PHSM) should apply a standard methodology such as the *WHO rapid risk assessment of acute public health events* [21]. *The WHO mass gathering COVID-19 risk assessment tool—Generic events* [21] provides such a standard methodology and structure for authorities and event organizers planning mass gatherings during the current COVID-19 pandemic.

Working amongst various NGDO and government partners, the Ascend West and Central Africa programme quickly adapted in order to navigate the uncertainty of the pandemic in March and April 2020. Countries wishing to restart NTD activities could be accommodated by a process that gave them the best chance of doing so successfully, whilst at the same time “flexing” the programme to focus on developing and disseminating COVID-19 prevention-related Behaviour Change and Communication (BCC) materials. By adapting an existing WHO risk assessment tool for mass gatherings, the Ascend West and Central Africa programme was able to specifically address key considerations for NTDs through the creation of the Risk Assessment and Mitigation Action (RAMA) tools for the resumption of MDA, surgery, and surveys. These tools ensured that countries would identify COVID-19-related risks and install proficient mitigating actions.

Sightsavers and partners have adapted the generic tool and methodologies to specifically address key considerations for neglected tropical diseases. This includes three risk assessment and mitigation action (RAMA) tools:

- RAMA I: Distribution of treatments.
- RAMA II: Active case finding and surgical outreach for trichiasis and hydrocele surgeries.
- RAMA III: Population-based surveys.

**Fig. 1:**
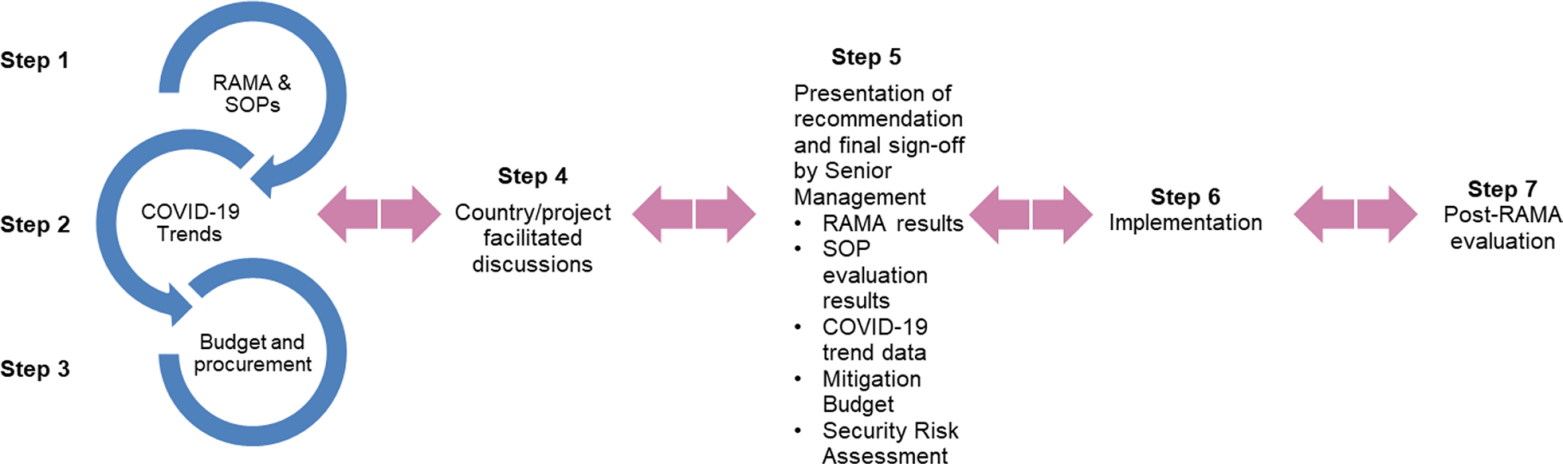
Example: RAMA tool as part of a comprehensive approach to the resumption of NTD activities. *RAMA* risk assessment and mitigation action, *SOP* standard operating procedures, *COVID-19* coronavirus disease 2019

In line with WHO guidance [20–22], adjusting any Public Health and Social Measures (PHSM) should be considered at the sub-national level starting in areas with lowest incidence of COVID-19. Where feasible, resumption of activities should be conducted in a controlled, measured, and stepwise manner. To ensure that the resumption of NTD activities is undertaken as safely as possible, ascend recommends that national NTD programmes and partners use the RAMA tools as part of a more comprehensive seven stage risk management approach (Fig. 1) to ensure compliance with the WHO guidance, including close monitoring of COVID-19 trends, independent verification and evaluation of written standard operating procedures (SOPs), confirmation of mitigation budget and availability of any required personal protective equipment (PPE), and completion of additional security risk assessments. Upon conclusion of the planned activity an updated COVID-19 trend report is submitted, along with results of routine monitoring and evaluation of activities. As a result of this process, since July 2020, seven separate requests to resume NTD activities have been processed and activities successfully completed, including MDAs and disease specific assessments supported under the Ascend, Accelerate and GiveWell programmes. Many more requests have been approved and are under consideration showing that there is a clear pathway for NTD activities to resume safely despite the uncertain context of COVID-19.

## Behaviour change messaging: ascend project experiences in West and Central Africa

The far-reaching and hard-hitting implications of the COVID-19 pandemic struck at a time when the Ascend West NTD programme was nearing the end of its first year of activities. A contingency plan was developed which included identifying a set of opportunities where Ascend competencies, experience, and ability to innovate in contributing towards the COVID-19 response efforts was developed. To formulate the country proposals extensive collaborations were required across the consortium, with ministries and partners.

**Fig. 2:**
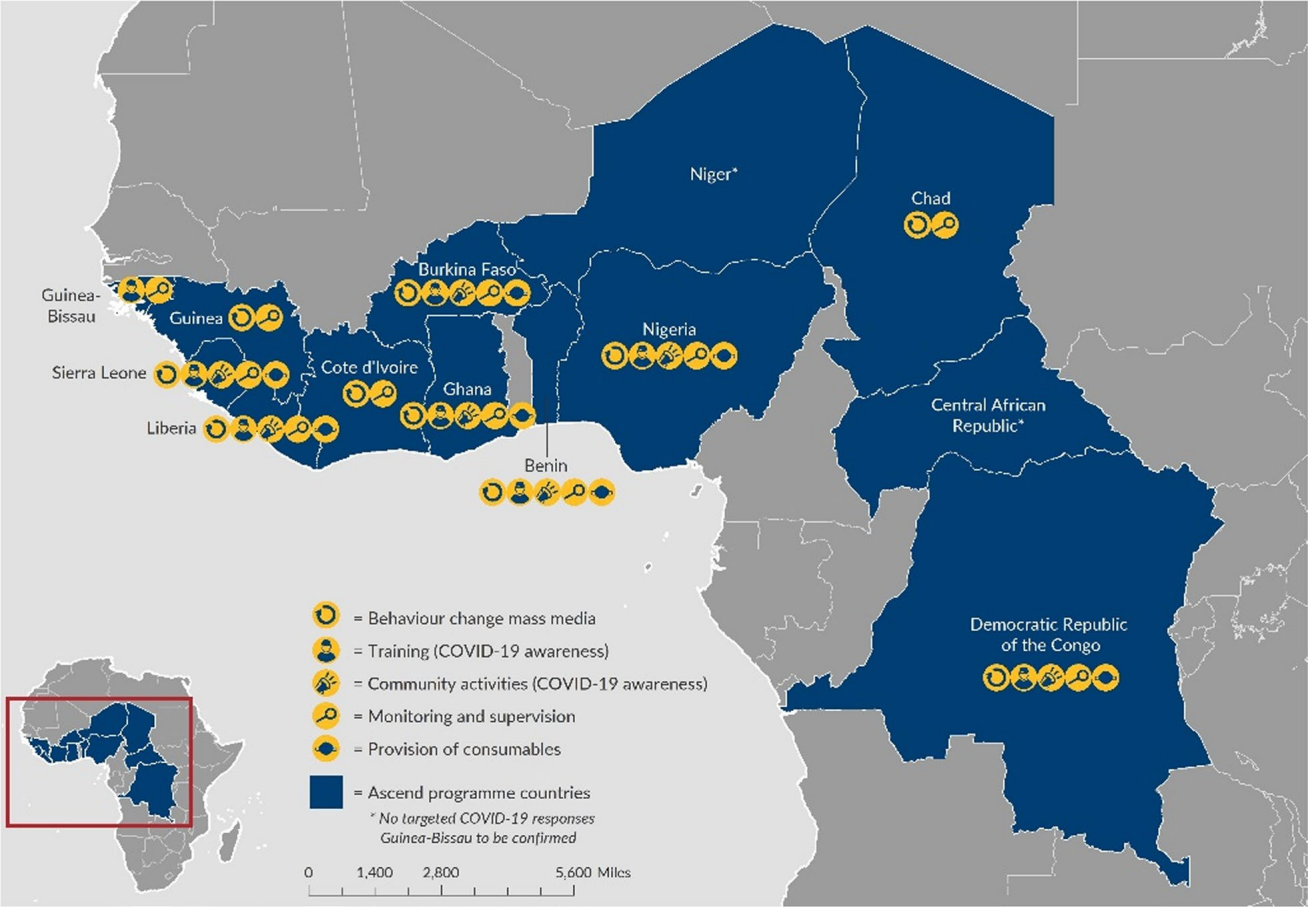
Map showing activities underaken in Ascend West and Central Africa’s Covid-19 response in 2020

Risk communication and behaviour change was a common theme requested by Ministries from 10 of the countries (Benin, Burkina Faso, Chad, Cote d’Ivoire, DRC, Ghana, Guinea, Liberia, Nigeria, Sierra Leone). The activities for each country were unique and designed to fit with the need in country at that time (Fig. 2).

## Process

The process for developing the communication campaigns started with scoping sessions. It was necessary to understand where the need for communication materials was in terms of geography and demographics, protective behaviours and communication channels. Discovery sessions were then held with the Ministries, where Ascend teams learnt about communications that had been developed to date, their successes, their shortfalls and then discussed what may be required in terms of Social Behaviour Change and Communication (SBCC) materials. These conversations were then distilled into scopes of work for each country, which included a recommendation on media channels, what quantity of materials would be required, what languages should be used and also whether accessibility adapts would be required e.g. captions on videos or the inclusion of signers.

In conjunction with the scoping exercise, we developed a communications strategy for each country, using key local insights from the Ministries of Health and in-country teams. This strategy reflected the key messages and protective behaviours upon which the Ministry wished to focus. Three strategic territories were developed—*Truth, Play your Part and Protection*. We found that although each of the countries had unique challenges and barriers there were some common “universal truths” in all countries.

After receiving approval on the strategy and territory the local agency was briefed in each country using the insights we had gleaned from the process to date. The local agencies then proposed with different creative routes that could answer our brief, each pulling on different emotions and cultural truths and all seeking to increase motivation to uptake protective behaviours. These concepts were then presented back to the Ministry for their input and feedback. It was at this stage teams were able to focus on how we could make the communications as inclusive as possible. Considerations were made for literacy, legibility and representation within all the communications. Revised creative feedback was presented to the Ministries up to three times before the production was commenced including filming, photography, illustration, and copywriting.

## Outputs

The communications campaigns across the nine countries with M & C Saatchi World Services support include a wide variety of creative assets. This includes television commercials, radio spots, billboards, banners, training guides, fliers, training videos, influencer videos and social media assets. The expected reach is over 100 million people which based on projected figures of the estimated the cumulative audience size of each media.

These figures will be supplemented with a phone survey to 300 participants in each country that will be conducted by Geopoll, a third party research company. This survey will measure impacts such as awareness, motivation to take action, after hearing the advertising materials, and some softer measures from beneficiaries around resonance and relevance.

## Key challenges and successes

A key challenge in this process was establishing who was responsible from within the Ministries to take part in the conversations and who had authority and the ultimate sign off on any decision. It is necessary to have the people that are making decisions present in the (virtual) room; without engagement from these key stakeholders early in the process sign off later could be problematic and time consuming. The successes became apparent when the Ministry identified a specific audience for communications during the initial discovery sessions which enabled the campaigns to be tailored almost entirely to their needs and cultural nuances.

**Fig. 3:**
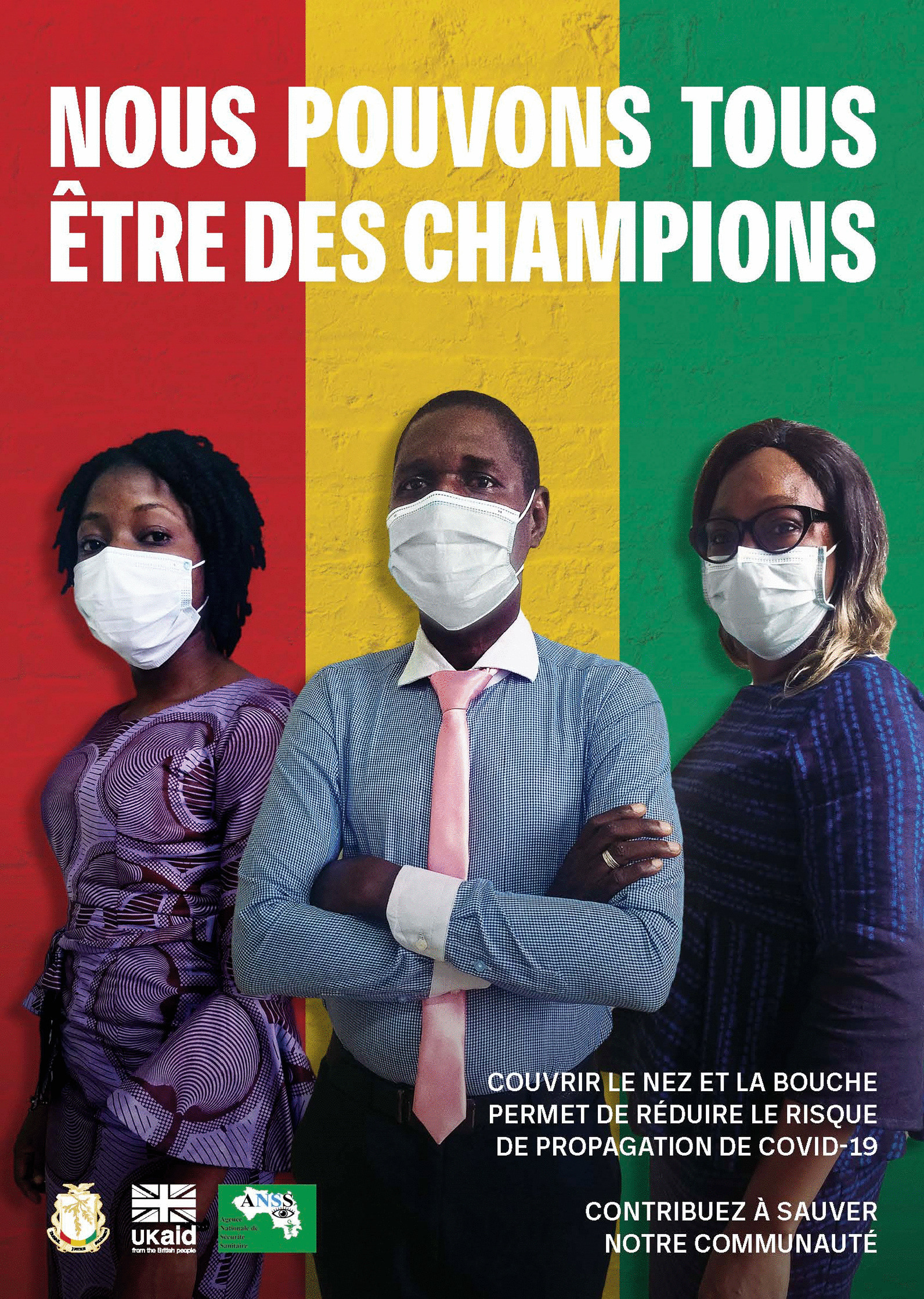
Guinea poster. Poster created for ANSS and Health Service in Guinea, headline reads ‘We can all be champions’ by covering your mouth and nose you can stop the spread of COVID-19

**Fig. 4:**
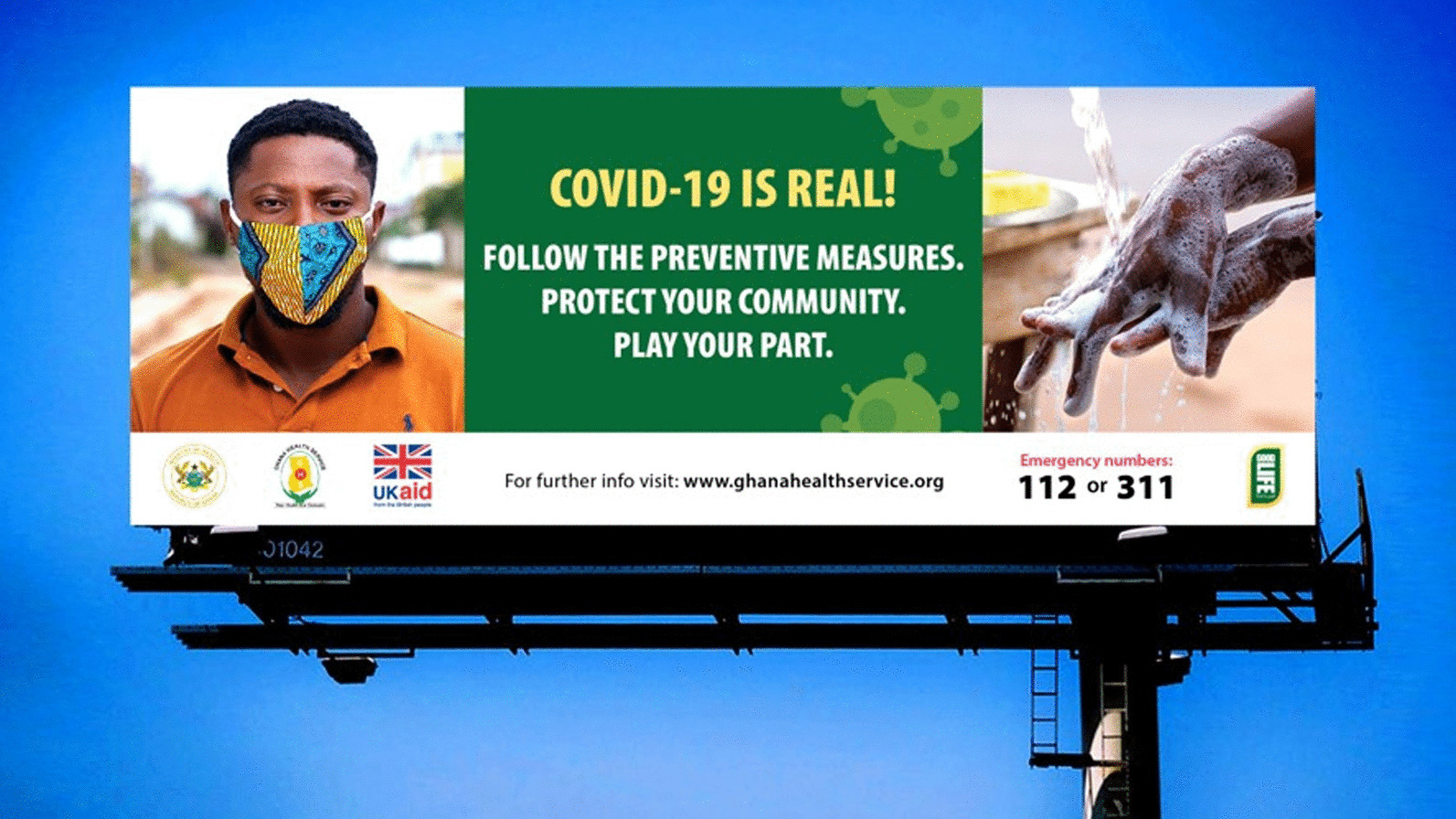
Ghana OOH in situ. Ghana billboard created for Ghana Health Service and Goodlife. Dispelling misinformation by addressing the rumour that COVID-19 was not real, head on; and showing what the protective behaviours look like

**Fig. 5:**
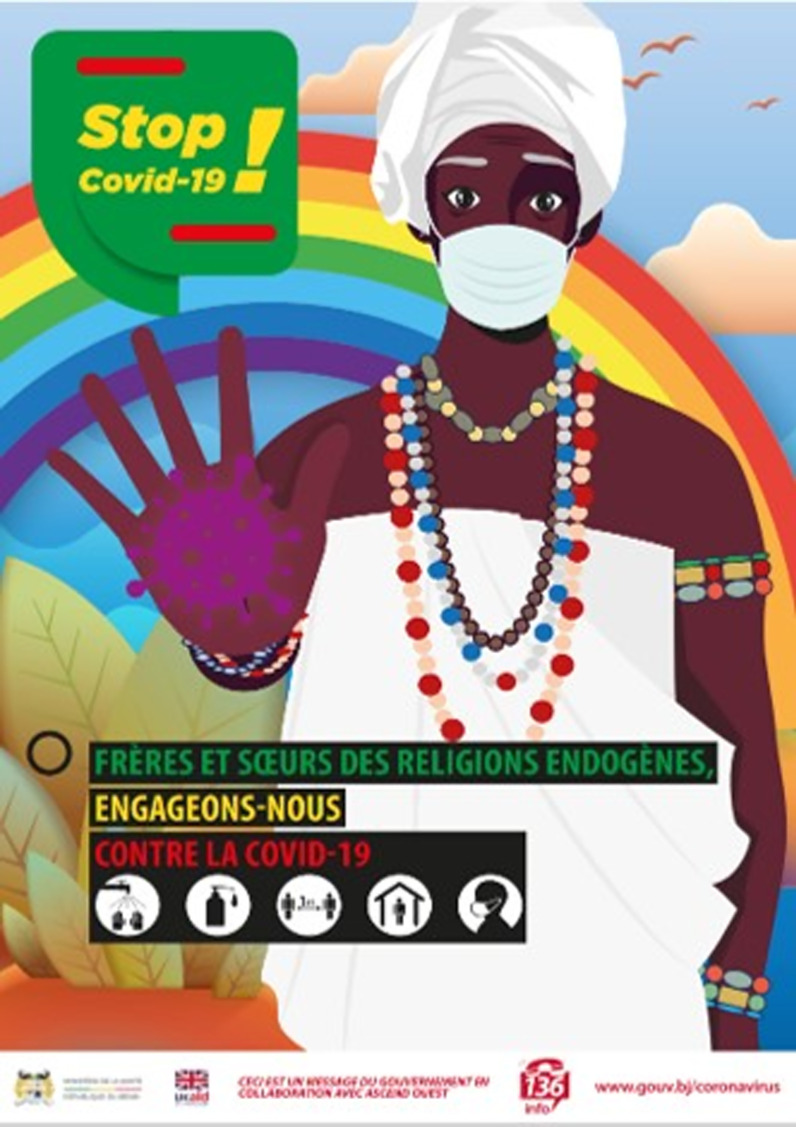
Benin religious leader guide. Front page of a guide made for endogenous religions, informing them on how to direct their communities and themselves to stop the spread of COVID-19. Two additional guides were made for Christians and Muslims and distributed through community volunteers

**Fig. 6:**
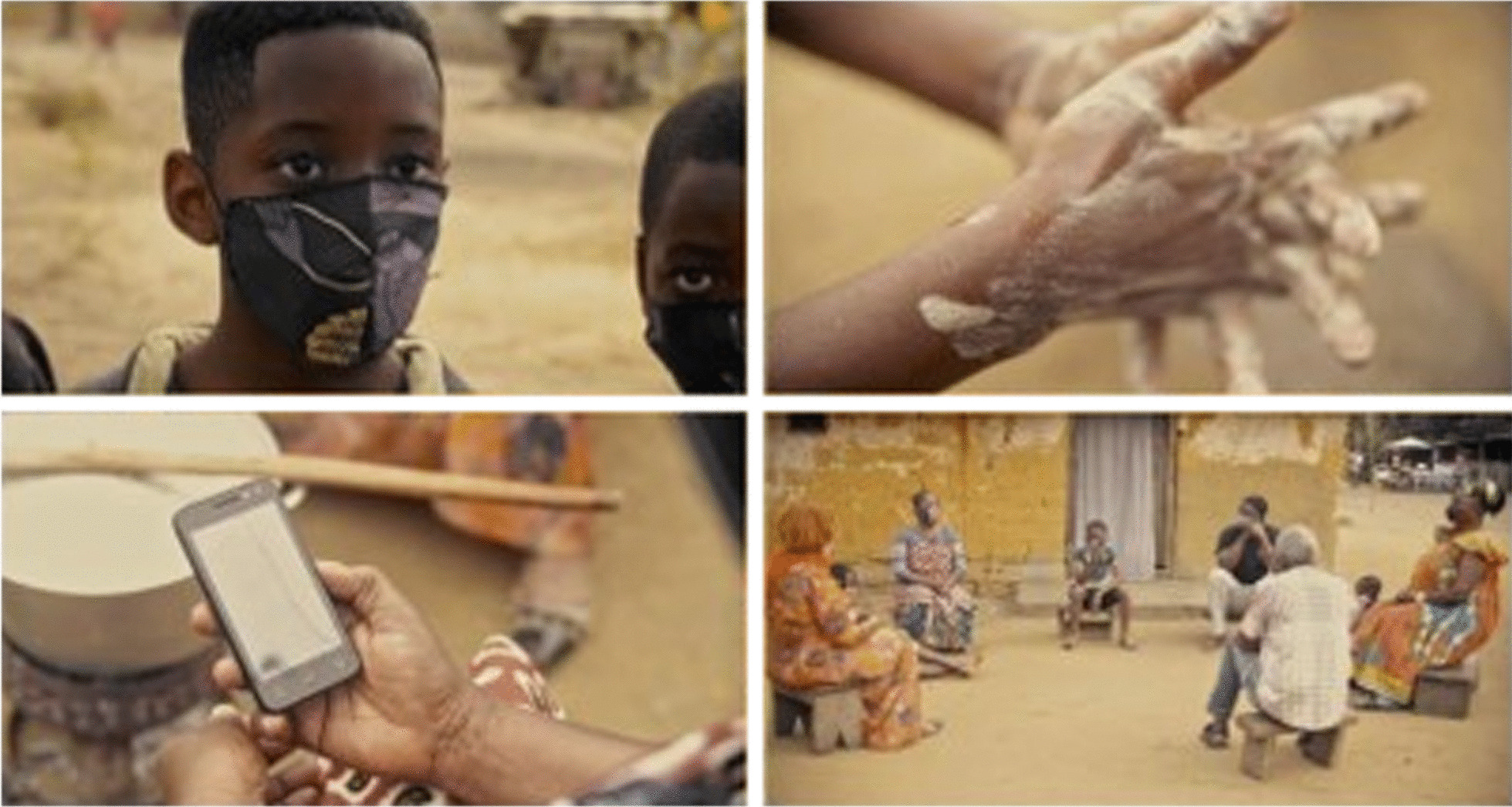
Democratic Republic of Congo (DRC) TV stills. Still images from Lingala TVC ‘Telemela’ (meaning stop in Lingala), created for Health Service in DRC. Using popular actress and comedian Mere Bipendu to teach young children and the wider community, in the rural provinces, how to protect themselves from COVID-19

Selected figures of the material developed are shown in Figs. 3, 4, 5 and 6.

## Tentative time horizons: looking into the crystal ball

### The short term

In the short term, unless MDA and morbidity management activities resume, achievement of NTD targets as projected in the new WHO/NTD Roadmap (2021–2030) will be deferred, the aspirational goal of NTD programmes to enhance UHC will be jeopardised, essential medicines will not be delivered to the poorest and ‘leave no one behind’ will become a hollow call. Any significant delay may require additional rounds of MDA or the consideration of twice-yearly MDA which will involve significant cost increases but initial baseline prevalence, historic coverage and the disease and its epidemiology will be the determinants. Resumption of MDA with the necessary risk assessment process in place is showing that an important health intervention can be restarted at limited risk and with potentially huge benefits.

Failing to complete MDA will deprive populations of essential drugs and, ironically, leave them further behind! Sightsavers, as a key player in the NTD sphere, and prominent in the Accelerate and Ascend West and Central Africa programmes, has already published documents on the COVID-19/NTD scenarios [1, 15].

The organization has already collaborated widely with countries to develop risk assessment and mitigation action (RAMA) tools for national NTD programme managers and partner/donor representatives to assess together the specific risk of COVID-19 on the delivery of NTD treatments and to determine the overall risk of spreading the virus with specific mitigation measures in place (see above).

With the RAMA tools we have shown the COVID-19 crisis need not allow the major health issues that confront poor and vulnerable populations to be forgotten. Indeed, the resilience of health interventions, exemplified by NTD projects, to manage and implement integrated disease control responses should be the model for progress compatible with other public health programmes and interventions. We, therefore, show a reassessment of the status of NTD activities to ensure that within the limits of practicable safe approaches, activities can be reinstated. For example, in rural settings where MDA for NTDs has been a critical driver of success through strong community engagement, providing access ‘beyond the end of the road’ to many millions distant from formal health services. NTD/MDA and seasonal malaria chemotherapy (SMC) and immunisation programmes can work together to ensure the most vulnerable are not deprived of access to life-saving interventions.

The resumption of these activities will have a dramatic impact, in particular, for SMC and children’s immunisation, decreasing risk of mortality in a group less vulnerable to the impact of COVID-19 and calm any knock-on effect to the current public health crisis. However, there will be increased costs associated with the resumption of MDA as well as increased costs of safer surgery (trichiasis and hydrocele) to reduce COVID-19 risks. These are currently estimated to be of the order of 30%—a cost that must be weighed against the benefits derived for those currently living with vision impairment, stigma and disablement. The risk of epidemic measles outbreaks can be predicted if immunisation programmes are not speedily resumed with high mortality ensuing; outbreaks in several African countries with significant mortality were reported by WHO in late 2019 [23]. As we have already stressed, implementation of MDA by communities via community-directed distributors (CDDs) can be a platform for COVID-19 mitigation messaging while providing populations access to free medicines included on the WHO Essential Medicines List. CDDs have historically had a high proportion of women acting as MDA distributors empowered by their own communities [23].

Reassessment of country situations will be an ongoing process as COVID-19 continues to have an impact on Africa. The virus is now endemic, and governments will need to adapt and balance public health priorities with social, political and economic impacts. Suspending health programmes will have a greater detrimental impact on health than COVID-19 in the long term, particularly on children. Implementation of NTD and other programmes with their extensive community reach and engagement with vulnerable populations is compatible with COVID-19 control as articulated in previous Sightsavers documents [24] https://www.sightsavers.org/news/2020/06/water-sanitation-hygiene-strategies-takling-covid-19/ and https://www.sightsavers.org/programmes/ascend/.

Programmes must learn to adapt strategies to COVID-19 and plan for the medium to long-term. Partners will continue to articulate that MDA suspension blocks an opportunity for the delivery of major interventions relevant to the achievement of UHC—the distribution of essential medicines—which when managed safely can benefit and mitigate COVID-19 impact. This applies, particularly, to the most vulnerable individuals. Preventing access to surgery to address disabling conditions (e.g. trichiasis and hydrocele surgery) prevents the opportunity for the resumption of productive lives.

### The medium term

Countries and the global health community will have better appreciation of the real impact of COVID-19 on different regions, communities (urban, peri-urban, rural, migratory populations) and geographies of Africa and of the impact of lockdowns on the spread of the virus; we reiterate that one size does not nor will not fit all scenarios [1]. The impact of any reduction in immunisation coverage will become evident with potential increases in measles cases; similarly, it will be important to evaluate any change in malaria cases and under-five mortality. Greater coordination and cooperation between NTD and other programmes could be a positive impact of COVID-19 on the health system itself [9]. The broader impact on the human resources of the health systems and where the critical capacity is needed will also be evident. ‘Hotspot’ countries and localities within countries at the highest risk will have been identified where specific mitigation measures might be necessary following any suspension of MDA [25].

### The longer term

It is likely that the acute impact of COVID-19 will have passed but that the implications of political decisions will be clear and the new normal of a new endemic virus—the ‘chronic COVID-19 pandemic’ recognised. Given the fragility of many African health systems and the human resource and knowledge constraints, planning a way forward will be necessary. The NTD community has strong foundations of skilled personnel within countries who can show the necessary leadership qualities to address this new environment. Such leaders can assert that failing to deliver interventions that address the needs of the poorest is contrary to human rights law, is unethical, and fails to recognise their benefits in COVID-19 mitigation. The NTD community reach and the ‘beyond MDA’ supplementary interventions (hand/face washing/limb care, sanitation) are powerful COVID-19 mitigating factors readily applicable and presently receiving donor support.

## Discussion

We categorise what we believe are the “unknowns” and “knowns” in the current COVID-19 context. The unknowns are the true case numbers of COVID-19 in Africa and the excess deaths associated with COVID-19. Given the variable quality of surveillance, the extent and effectiveness of testing as well as the cause of death and the paucity death reporting, particularly in rural settings, the true situation cannot be known. This is despite the suggestion that to date, COVID-19 has not impacted on Africa as might have been expected. We are unsure of how rapidly COVID-19 will spread given the different national responses to easing lockdown and in different African eco-geographies—one size is unlikely fit all scenarios.

The impact the focus of health authorities on COVID-19 will have on those most vulnerable in Africa, particularly children under five years of age (Under 5 s) if programmes are suspended or delayed with potential increases in Under five mortality remains to be elucidated. It will take several months to have any data to make a judgement but polio eradication activities, malaria chemotherapy programmes, immunization programmes and NTD programmes demand an equal case for speedy resumption of activities.

We do not know if there will be a different impact given median age of African population (circa 20) being lower than in Europe (45) [26] for example: will there be higher proportion of asymptomatic carriers compared with Europe? In addition, will there be different ‘underlying health conditions’ in Africa compared with more developed societies, and will there be a different clinical impact on COVID-19 patients given the potentially high level of undiagnosed diabetes and hypertension in urban areas? An important question is are people with disabilities—with visual impairments, hearing impairments, physical disabilities, and mental health conditions—being considered within the COVID-19 response as highlighted by the UN Secretary General’s Report [27]. We do not believe that these unknowns preclude the known benefits of resuming safety assured NTD activities. The benefits of delivering donated essential medicines to the poorest outweigh the risks given the “hybrid” benefits to the elimination and control of NTDs on the one hand, and mitigation against the COVID-19 chronic pandemic on the other given the commonalities of impact on transmission of water, sanitation and hygiene associated interventions and behaviours.

However, we know that COVID-19 will behave biologically in the same way in Africa as elsewhere in terms of infectivity and modes of transmission; MDA activities can resume as soon as possible with the requisite safety measures in place for distributors and communities following risk assessment. Mitigating measures, including WHO guidance that *at least* one metre social distancing is recommended, although national policies may recommend two metres, a norm for behaviour in most organized and socially responsible societies globally.

There will be increased costs to deliver MDAs safely as well as to carry out surgeries. More time will be needed so resources may need to be redeployed. However, as NTD programmes address the most vulnerable, disadvantaged and those with disabilities—the so called “bottom billion”—there is an ethical and human rights element to activate programmes. MDA prevents chronic disabilities as well as providing other synergistic health benefits; hence, resumption of activities is a health as well as a development pre-requisite in the broader context of the sustainable development goals (SDGs). NTD programmes reach beyond the ‘end of the road’ via community-based volunteer personnel with a proven track record of delivery—resources all of which can be harnessed now. NTD MDA in the COVID-19 era will provide an opportunity to enhance efficiencies through better inter-programme coordination, particularly with malaria and immunisation programmes but also as pointed out in [9] through greater sectoral interaction.

A principle of public health and medicine is to “do no harm” and “leave no-one behind”. These tenets will be jeopardised by a failure to resume MDA safely and suspend morbidity management as both platforms can enhance a country response to the COVID-19 pandemic. The value of other NTD interventions (intensified handwashing practices, sanitation, safe water provision) which reduce transmission of both COVID-19 and NTDs are compatible health messages whilst NTD programmes also address and have experience in managing cross-border issues. For the reasons and experience we illustrate we recommend this “hybrid” approach be a policy priority.

## Conclusions

COVID-19 is now an endemic infection to which all health programmes will be required to adapt against a background of uncertainty into the foreseeable future.

In the context of NTD programmes particularly in Africa, we consider the additional risks of resuming NTD interventions in areas where routine health services are ongoing can be mitigated, negating additional risk. In doing so we must be reassured that programmes and activities do no harm, requiring risk analysis and investment in mitigation measures, despite increasing costs, given the biggest cost driver will be additional time of human resources required, a conclusion borne out by the ongoing Risk Assessment and Mitigation Actions (RAMA process).

NTD programmes offer an opportunity to reinforce COVID-19 response through health-impact messaging. The NTD community must address this because any increased costs offer a potentially wider impact through ‘hybrid’ interventions thus being more cost-effective. Such an approach facilitates the strengthening health systems, facilitating integration and cross-sector collaboration.

Having initiated COVID-19 flexing policies in Ascend West countries we are now better informed about the new context within which NTD interventions will reside. The situation will evolve over time, unpredictably and according to country circumstances. This evolution will not be driven by COVID-19 biology but by national policy responses to mitigate impact and societal adherence to behaviour change messages to stem transmission.

## Data Availability

Access to photographic images of behaviour change materials can be made available through the authors via the corresponding author.

## Abbreviations

APOC: African programme for onchocerciasis control
CDDs: Community directed distributors
COVID-19: Disease due to SARS-CoVAR-2
ESPEN: Expanded special project for eliminating neglected tropical diseases
LMIC: Low and middle income countries
MDA: Mass Drug Administration
NTDs: Neglected tropical diseases
ODA: Official development assistance
RAMA: Risk assessment and mitigation
SMC: Seasonal malaria chemotherapy
SOPs: Standard operating procedures
SDG: Sustainable development goals
UNCTAD: United Nations conference on trade and development
UHC: Universal health coverage
WHO: World health organization
